# Correlation between β-catenin mutations and expression of Wnt-signaling target genes in hepatocellular carcinoma

**DOI:** 10.1186/1476-4598-7-21

**Published:** 2008-02-18

**Authors:** Madeleine Austinat, Ruediger Dunsch, Christian Wittekind, Andrea Tannapfel, Rolf Gebhardt, Frank Gaunitz

**Affiliations:** 1 Institute for Biochemistry, Medical Faculty, University of Leipzig, Johannisallee 30, 04103 Leipzig, Germany; 2 Institute for Pathology, Medical Faculty, University of Leipzig, Liebigstrasse 27, 04103 Leipzig, Germany; 3 Institute for Pathology, Ruhr-University-Bochum, Berufsgenossenschaftliche Kliniken Bergmannsheil, Bürkle-de-la-Camp-Platz 1, 44789 Bochum, Germany

## Abstract

Aberrant Wnt-signaling caused by mutants of β-catenin, a key regulator of the canonical Wnt-signaling pathway, is frequently detected in cancer. Only recently, it was suggested that in hepatocellular carcinoma (HCC) the expression of the target gene glutamine synthetase (GS) is a highly reliable marker for the identification of β-catenin mutations. In order to prove this hypothesis, 52 samples from human hepatocellular carcinomas were analysed for the activation of β-catenin and the expression of GS. In total, 45 samples stained positive for cytoplasmic/nuclear β-catenin. A strong correlation between expression of GS and activated β-catenin (100% of nuclear and 84% of cytosolic) was found. However, among 35 GS positive tumors that were analysed for β-catenin mutations no mutations were detected in 25 GS-positive carcinomas although 24 out of the 25 carcinomas exhibited at least abnormal expression of β-catenin. Since the mutational analysis identified 9 different point mutations of the β-catenin gene including the rare mutation H36P and the yet unknown mutation P44A it was asked whether these mutations may differently effect β-catenin target genes. Therefore, expression plasmids for different mutations were constructed and cotransfected with the TOP-flash luciferase reporter and a reporter carrying the GS-5'-enhancer. The experiments confirmed that there are differences between different β-catenin target sequences and different β-catenin mutations. In addition, the failure that the endogenous expression of GS in GS-negative cells was not induced by the transient transfection experiment indicated that the effect of β-catenin on the GS-5'-enhancer is only one aspect of gene activation induced by β-catenin.

## Background

Hepatocellular carcinoma (HCC) is the most frequent cancer worldwide (for review see [1,2]). During the last years it became obvious that active Wnt-signaling as characterized by the presence of nuclear/cytosolic β-catenin highly correlates with the occurrence of HCC in animal models and in human patients [3-6]. Under normal conditions the activated (nuclear) form of β-catenin is a transcriptional activator mainly involved in the regulation of cell proliferation, differentiation and stem cell maintenance [7-9]. As a transcriptional regulator β-catenin interacts with members from the LEF-1/TCF transcription factor family, probably by removal of co-repressors like groucho [10-12]. It should be noted that LEF-1/TCF is an architectural transcription factor that upon binding to its target sequence together with corepressors and in the absence of β-catenin inhibits expression.

In differentiated, non-proliferating cells, β-catenin is associated with membrane bound E-cadherin [13] and non-bound molecules are quickly removed from the cytosol in the absence of Wnt-signaling, thereby preventing its translocation to the nucleus. This is accomplished by a multienzyme complex that binds cytosolic β-catenin. When bound to the complex β-catenin is phosphorylated by active glycogen synthase kinase 3β(GSK3β), then labelled by ubiquitinylation and finally degradated by the proteasome. The complex, in addition to GSK3β, contains other proteins like Axin1, Casein Kinase Iα (CKIα) and APC (Adenomatous Polyposis Coli protein) (for review see [14]). Although mutations in each of the components of the complex may cause abnormal cytosolic stabilization of β-catenin, mutations of the β-catenin gene itself appear to be the most common cause for stabilization in pathological situations. These mutations usually affect residues at position 33 (S), 37 (S), 41 (T) or 45 (S) located in exon 3 of the human β-catenin gene that are the targets of priming phosphorylation by CKIα (S45) or subsequent phosphorylation by GSK3β (S33, S37 and S41). Activation of β-catenin finally leads to the transcriptional activation of a variety of genes [15]. However, not much is known about the role of specific genes activated with regard to their role in tumor development. On the other hand, glutamine synthetase (GS), one of the enzymes identified to be regulated by nuclear β-catenin, may be a candidate that contributes to enhanced malignancy of HCCs [16,17]. In fact, Osada *et al*. demonstrated that GS expression may enhance the metastatic potential in HCC, and that GS immunostaining identifies HCC patients with a high risk for disease recurrence after curative hepatectomy [18]. In the intact liver, GS is confined to a small population of hepatocytes located around the hepatic terminal venules and is regulated in a highly complex manner [19]. With regard to hepatocyte specific expression, several regulatory elements have been described, including 5'-upstream elements [20-22] as well as intronic elements [23,24]. Recently published experiments identified a putative binding site for members of the LEF-1/TCF transcription factor family in the main 5'-enhancer that may be responsible for activation of transcription by activated β-catenin [22]. However, it is not known whether the 5'-enhancer does mediate enhancement of expression in the presence of activated β-catenin. Therefore, we investigated human HCCs for activated β-catenin and specific expression of GS and we identified different mutations of exon 3 of the β-catenin gene. Finally, we were able to demonstrate that different mutations of β-catenin are able to activate expression from reporter genes with the 5'-enhancer of the GS gene.

## Results

### Immunohistochemical detection of β-catenin and glutamine synthetase in hepatocellular carcinomas (HCCs)

**Figure 1:**
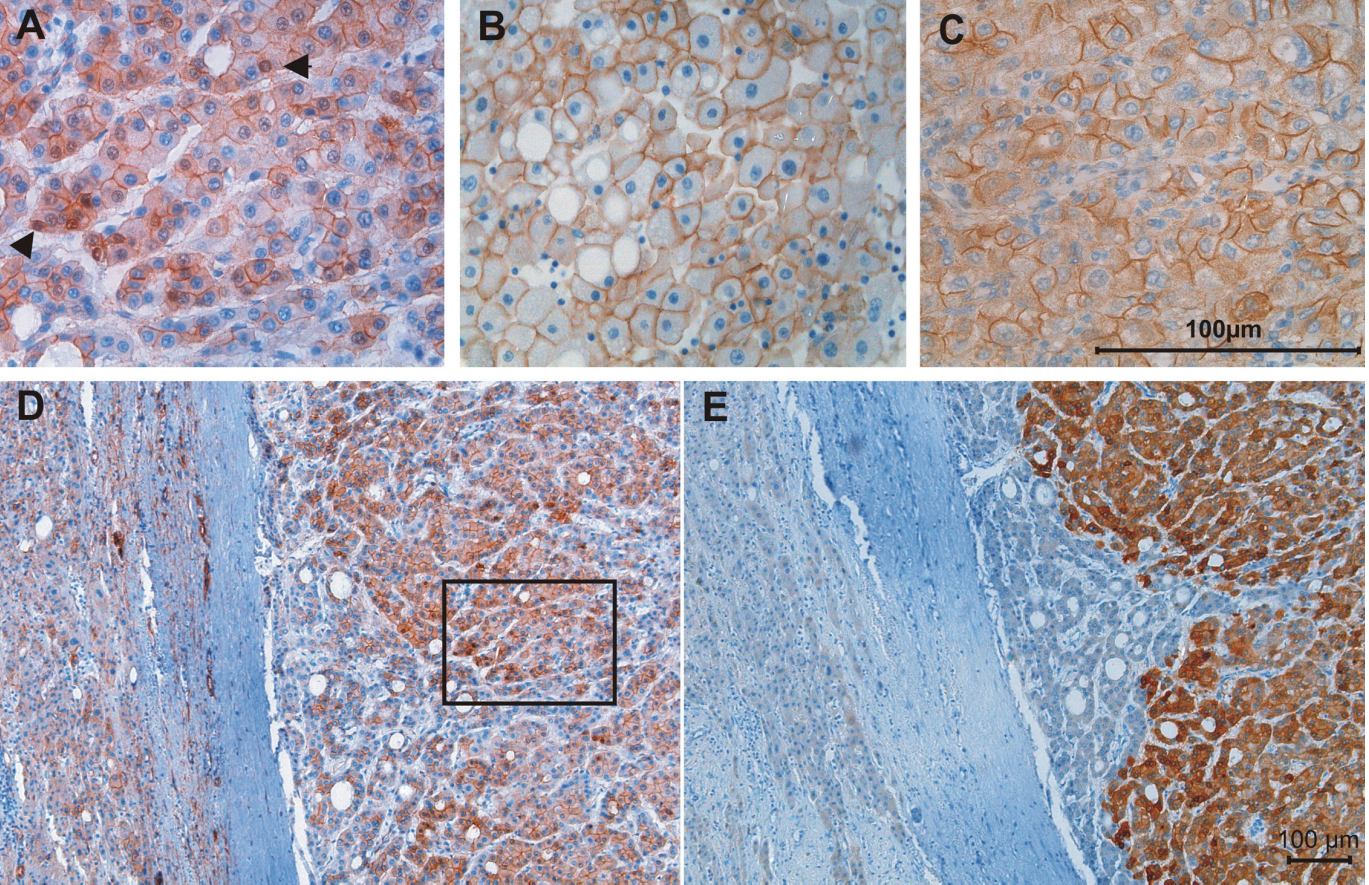
**Examples of β-catenin phenotypes detected in human hepatocellular carcinomas.** A: Magnification of the squared area seen in 'D' with β-catenin located in the cytoplasm and also in several nuclei (arrowheads). B: Tumor with normal confinement of β-catenin to the cell membrane and C: cytoplasmic staining of β-catenin without detectable expression in the nuclei. D and E: serial sections of an HCC expressing glutamine synthetase (E) and nuclear β-catenin (D). Original magnification: 400× (A, B, C) and 100× (D, E). The mutation of β-catenin in the serial sections was S33C.

**Figure 2:**
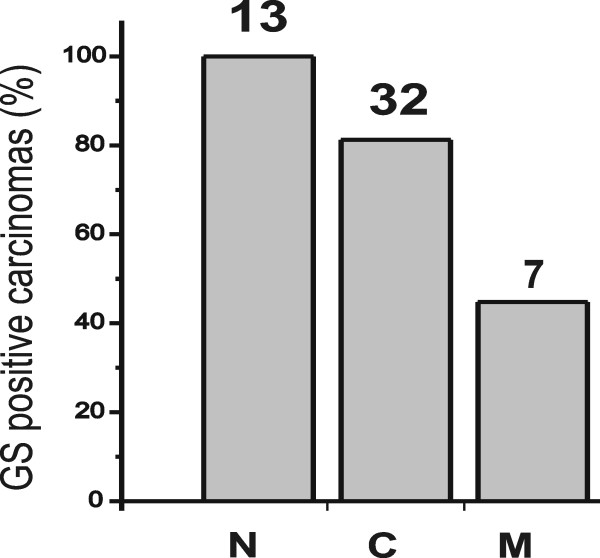
**Correlation between expression of GS and the phenotype of β-catenin expression.** The %age of GS-expressing tumors with nuclear (N), cytosolic (C) and normal membranous (M) β-catenin expression is indicated. Numbers on top indicate the total amount of HCCs in each group.

In a first series of experiments 52 human tumor samples were analysed for the expression of GS and β-catenin. Immunohistochemistry identified β-catenin positive nuclei in 13 specimens (e.g. Fig. 1A/D). Cytoplasmic but no obvious nuclear staining was detected in 32 tumors (e.g. Fig. 1C) and normal membranous expression of β-catenin was observed in 7 HCCs (e.g. Fig. 1B). All tumors that exhibited a nuclear presence of β-catenin were expressing GS (e.g. Fig. 1D/E). In contrast, the cytoplasmic presence of β-catenin was associated with GS expression in 84% of the HCCs but still 43% of the HCCs with normal membranous β-catenin staining were GS positive (Fig. 2).

### Analysis of β-catenin mutations present in GS-positive hepatocellular carcinomas

Next it was asked, whether the phenotypes observed by immunohistochemistry were associated with certain mutations of the β-catenin gene (*CTNNB1*). Therefore, DNA was extracted from samples, amplified by PCR and sequenced. It was possible to amplify DNA from 40 samples out of the 52 specimens that were investigated by immunohistochemistry. The analysis revealed 9 different point mutations of the *CTNNB1 *gene in 10 (25%) patients (Table 1). All mutations affected the degradation box in exon 3 of β-catenin. In 4 cases residues for GSK-3β phosphorylation were changed, namely to cysteine (S33C, S37C), to phenylalanine (S37F) or to alanine (T41A). Furthermore, the mutations I35S and H36P (two cases) were identified within the β-TrCP recognition sequence. Serine at position 45 was replaced by proline in two cases and to phenylalanine in 1 case. One of the patients with S45P exhibited a mutation of proline 44 to alanine in addition. In 8 out of the 10 samples with mutations we observed nuclear accumulation of β-catenin. The two cases of GS positive tumors without definite nuclear staining for β-catenin (P44A and S45P) at least showed a cytoplasmic presence of β-catenin. Therefore, it is obvious that every specimen with mutations of the *CTNNB1 *gene exhibited expression of GS. However, no mutations within the degradation box in exon 3 of the β-catenin gene were found in 25 GS positive tumors, 20 of them expressing cytoplasmic, 4 nuclear and 1 normal, membranous β-catenin (for complete set of data see Table 2).

**Table 1:** β-catenin mutations detected in HCCs. Indicated is the number of the codon affected and the resulting amino acid change as well as the phenotype observed by immunohistochemistry with regard to presence of β-catenin (cytoplasmic/nuclear) and GS-expression (GS).

Patient number	Codon	Mutation	Aminoacid	β-Cat cytoplasmic	β-Cat nuclear	GS
20071/2,3,-00	33	TCT > TGT	S>C	+	+	+
30600/4-05	35	ATC>AGC	I>S	+	+	+
334/2-02 24138/E-01	36	CAT>CCT	H>P	+	+	+
14865/2-05	37	TCT>TGT	S>C	+	+	+
4836/3-05	37	TCT>TTT	S>F	+	+	+
21217-00	41	ACC>GCC	T>A	+	+	+
6700/2.3.-05	44	CCT>GCT	P>A	+	-	+
6700/2.3.-05 29923/B-03	45	TCT>CCT	S>P	+	-	+
28356/2-05	45	TCT>TTT	S>F	+	+	+

**Table 2:** Correlation between mutations of the β-catenin gene, the location of β-catenin and the expression of GS. In the table all cases analysed by immunohistochemistry are listed. +GS: GS-positive;-GS: GS-negative; +M: with mutation;-M: without mutation; n.d.: Mutations were not determined due to lack of DNA; C: cytoplasmic, N: nuclear and M: normal membranous expression of β-catenin.

	+GS +M	+GS -M	+GS n.d.	-GS +M	-GS -M	-GS n.d.
C	2	20	4	0	2	4
N	8	4	1	0	0	0
M	0	1	2	0	3	1

Since activation of β-catenin can also be caused by mutations in other genes of the Wnt-signaling pathway, the APC-, the GSK-3β- and the β-catenin-binding domains of the *AXIN1 *gene of 18 patients were analysed. In fact, two samples were identified with a 3 bp deletion starting with nucleotide 289 deleting a tyrosine at position 97 within the APC-binding site of Axin1 (AF009674). However, only one of the samples expressed cytosolic β-catenin that interestingly also expressed GS. The other tumor sample showed membranous β-catenin and no obvious GS expression.

### Influence of β-Catenin mutations detected in hepatocellular carcinomas on the expression of reporter genes with the GS-5'-enhancer

**Figure 3:**
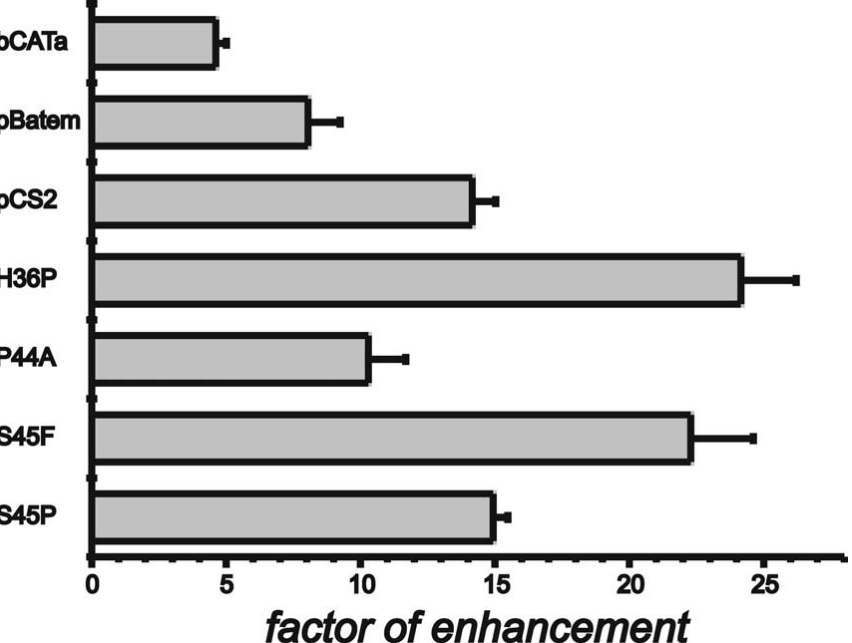
**Effect of different mutations of β-catenin on the expression of the reporter gene 'TOP-flash'.** Cells from the cell-line HuH7 were transfected with the reporter gene 'TOP-flash' harbouring LEF-1/TCF binding sites for β-catenin and the corresponding 'FOP-flash' without these sites. Each of the two reporters was cotransfected with several expression plasmids for different β-catenin forms: The construct 'bCATa' as a control not expressing any functional protein, the vector 'pBatem' expressing a truncated β-catenin, 'pCS2', expressing a form with a mutation of all N-terminal phosphorylation sites to alanine and in addition plasmids with mutations found in human HCCs: H36P, P44A, S45F and S45P. The expression from each co-transfection with 'TOP-flash' was compared to cotransfection with 'FOP-flash' in order to determine the factor of enhancement mediated by the mutants.

**Figure 4:**
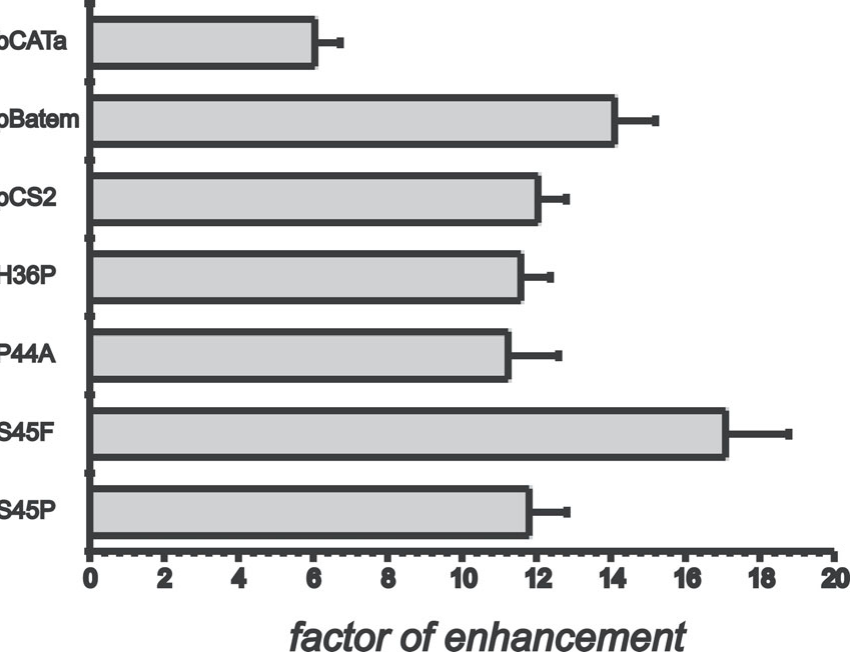
**Effect of different mutations of β-catenin on the expression of a reporter gene with the main 5'-enhancer of GS.** Cells from the cell-line HuH7 were transfected with the reporter gene 'HIII/EV_pT81' containing the main GS 5'-enhancer together with different expression plasmids for β-catenin forms (compare legend to Fig. 3). Expression was compared to expression from pT81 in order to determine the factor of enhancement mediated by the mutants.

Since we wondered whether the mutations of β-catenin detected in samples of human hepatocellular carcinomas are directly responsible for the activation of Wnt-target genes and especially for the expression of GS, plasmids were developed in order to express the mutated forms of β-Catenin in transfected cells. Therefore, we constructed expression plasmids, including ones for the rare mutation H36P and the non-described mutation P44A as well as expression plasmids for S45F and S45P. Additionally, reporter gene expression in the presence of these new constructs was compared to expression in the presence of plasmids carrying a truncated β-catenin, lacking the N-terminal phosphorylation sites ('pBatem') and a β-catenin with all four phosphorylation sites mutated to alanine, designated 'pCS2' (S33A, S37A, T41A and S45A). In order to normalize for endogeneous activity of Wnt-signaling the control plasmid 'bCATa' was used. Firstly, the reporter genes were cotransfected into HuH7 cells together with the 'TOP-flash' plasmid that harbours six LEF-1/TCF-binding sites. As control the same plasmids were cotransfected with Fop-flash that is equal to 'TOP-flash' but does not contain the LEF-1/TCF-binding sites. Forty eight hours after transfection cells were harvested and luciferase activity was determined. The factor of enhancement was determined by comparing 'TOP-flash' expression with expression from 'FOP-flash' for each of the different expression plasmids used. The result of the experiment is presented in Fig. 3. As can be seen, 'TOP-flash' and 'FOP-flash' differ in their level of expression even in the negative control (transfection with 'bCATa') by a factor of almost 5. This actually indicates that there is some endogenous activity of Wnt-signaling present in the HuH7 cells used in the experiment. However, in the presence of activating β-catenin mutations the difference between 'TOP-flash' and 'FOP-flash' becomes strongly pronounced. The highest response was obtained with the mutations S45F and the rare H36P, whereas gene activation of the truncated β-catenin (pBatem) was comparable to P44A, the newly found mutation. The substitution of all phosphorylation sites (pCS2) was comparable to S45P. Therefore the β-catenin mutations detected in GS-positive HCCs are regulators of the LEF-1/TCF target sequences of the TOP-flash plasmid. However, the question remained whether they are also able to activate GS via its 5'-enhancer that was previously shown to harbour an active LEF-1/TCF-binding site. To resolve this question an experiment was performed with a reporter gene containing the GS-5'-enhancer ('HIII/EV_pT81'). This reporter gene carries the *Hin*dIII/*Eco*RV-fragment from the rat GS gene encompassing the DNA sequence from position -2516 to -2146 upstream from the transcriptional start point that includes the 5'-enhancer complex [22]. HuH7 cells were cotransfected with this reporter gene or with the control vector pT81 containing only the reduced HSV-tk-promoter (used for the construction of 'HIII/EV_pT81'). For cotransfection, again, the expression plasmids for the different variants of β-catenin were used. The factors of enhancement presented in Fig. 4 were determined by comparing expression of 'pT81' to 'HIII/EV_pT81' in the presence of the different expression plasmids for the β-catenin variants. The factor of enhancement of expression of 'HIII/EV_pT81' compared to 'pT81' in the presence of the reference control plasmid 'bCATa' (Fig. 4) again indicates the HuH7 specific background activity of Wnt-signaling. which in this experiment was ~6 fold. However, all other cotransfections with the other β-catenin forms including the ones identified in the study presented, led to a factor of enhancement comparable to that obtained with the Top-flash system. In fact, only enhancement mediated by the mutation S45F was slightly but significantly higher than the other ones. This experiment for the first time clearly demonstrates that the mutant variants of β-catenin do directly target the GS-5'-enhancer and appear to be responsible for enhanced expression of GS in the presence of mutated forms of β-catenin.

Since reporter gene experiments clearly demonstrated that the GS-5' enhancer is a target for enhancement of expression mediated by mutated β-catenin, it was asked how the expression of endogenous GS is influenced by β-catenin mutants. Therefore, the expression plasmid for β-catenin with S45F that proofed to be most effective in reporter gene experiments was transfected into HuH7 cells and GS activity was determined. However, we were not able to detect any enhanced expression of GS between 24 and 120 hours after transfection (data not shown). The same result was obtained with cells from other lines as FAO or primary rat hepatocytes, no matter whether overall GS-activity of the transfected culture was determined or single cell determination of GS expression using specific antibodies was employed (data not shown).

## Discussion

### GS expression, β-catenin expression and mutations of β-catenin in HCC

The nuclear and/or cytoplasmic stabilization of β-catenin is recognized as a hallmark of an altered Wnt/β-catenin pathway [25]. At the time the present study was initiated, mouse models already had demonstrated a close correlation between the activation of β-catenin and the aberrant expression of GS in hepatocellular carcinomas [16,17]. Since it was asked whether this may also be the case in human hepatocellular carcinomas 52 tumor samples from patients were analysed for the coexpression of activated β-catenin as exemplified by the nuclear or the cytoplasmatic presence of the protein and expression of GS. Only recently, two equivalent studies were published by Zucman-Rossi et al. [26] and by Audard et al. [27]. Although some observations from our study are in agreement with these investigations there are differences that need to be discussed. For instance, Audard et al. report that 37% of tumors with β-catenin mutations they examined were without cytosolic/nuclear staining of β-catenin. However, in our study every mutation (10 patients) resulted in nuclear (8 patients) or cytosolic (2 patients) staining of β-catenin. More importantly, although the nuclear/cytoplasmic presence of β-catenin highly correlates with aberrant expression of GS the inverse relationship was not true, since we observed a number of cases of strong aberrant GS expression in the clear absence of β-catenin mutations. Therefore, our data does not support the opinion of Audard et al. that GS immunostaining is a trustable indicator of β-catenin mutations, although aberrant expression of β-catenin highly correlates with expression of GS. In addition, a strict interpretation of the data from Audard et al. would be that GS is not activated by wild-type β-catenin but only by mutant forms. However, this is in contrast to our investigation as well as that of Zucman-Rossi et al. who detected aberrant staining in several hepatocellular carcinomas also in the absence of mutations of β-catenin. At this point it should be noted that the mouse models of Colnot et al. [28] and of Benhamouche et al. [29] clearly indicate that the aberrant expression of non-mutated β-catenin caused by a loss of APC is accompanied by aberrant expression of GS.

With regard to mutations of the Axin1 gene we have identified a 3 bp deletion affecting the tyrosine at position 97 within the APC-binding site of Axin1 in two out of 18 analysed HCC. Only one of these two samples expressed cytosolic β-catenin and GS and the other did not express β-catenin nor GS. Therefore, loss of Axin1 function does not necessarily cause aberrant expression of β-catenin and GS in agreement with Zucman-Rossi et al. [26].

In conclusion, the diagnostic value of GS expression for the detection of β-catenin mutations or even for Wnt-signaling is doubtful, since tumors can be detected that express GS even in the absence of aberrant β-catenin expression and not every tumor with cytosolic β-catenin expresses GS.

### How is GS activated by β-catenin at the molecular level?

One important question of the work presented was to analyse how mutations of β-catenin are able to influence expression of GS. The cell line HuH7 was chosen for transfection experiments, since it had a low Wnt-signaling background activity compared with cells from the lines FAO or HepG2 (data not shown). Reporter genes with the GS-5'-enhancer and the 'TOP-flash' reporter plasmid were transfected together with expression plasmids for different mutated β-catenins into HuH7 cells. The strongest factor of enhancement (~17 fold with the GS-5' enhancer and ~22 fold with 'TOP-flash') was observed in the presence of the mutation S45F. Serine 45 is known as the priming position since its phosphorylation by casein kinase 1 (CKI1) is necessary for the subsequent phosphorylation of T41, S37 und S33 by glycogen synthase kinase 3β (GSK3β) that finally leads to ubiquitinylation and degradation of β-catenin by the proteasome. Therefore, from this point of view it was not surprising that a mutation of this position caused the highest effect. However, S45P affecting the same priming position was less effective in both, 'TOP-flash' and the GS-5'-reporter. Interestingly, this mutation did not result in nuclear staining for β-catenin (Table 1). With regard to the GS-5' enhancer, enhancement of expression was more or less equal between the mutations S45P, P44A, H36P, pCS2 (all phosphorylation sites mutated to alanin) or pBatem (N-terminal region with all phosphorylation sites deleted). Interestingly, the rare mutation H36P is a strong mutation although it does not directly involve a phosphorylation site. However, it is close to the phosphorylation site S37 and the introduction of a proline in close proximity may alter the structure of the protein in a way that interferes with phosphorylation. The same may apply for P44A, the new mutation detected in this work, that also does not directly affect a phosphorylation site. At this point it may be interesting to note that P44A appears to be a rather rare mutant since it was not found among the 60 mutants identified by Audard et al. When the effects of different mutations on 'TOP-flash' and the reporter with the GS-5' enhancer were compared, some interesting differences could be observed. For example the rare mutation H36P caused the same strong fold-enhancement of 'TOP-flash' expression as S45F. However, the reporter with the GS-5' enhancer responded more strongly to S45F than H36P. In addition, the mutation P44A enhanced expression from 'TOP-flash' not as strong as H36P while in case of the reporter with the GS-5' enhancer both mutations were equally effective. These different responses of 'TOP-flash' and the GS-5' enhancer to different β-catenin mutants may indicate that regulation of GS by mutant β-catenin may be different to regulation of typical LEF-1/TCF target sequences as presented by 'TOP-flash'. This is an interesting aspect that should be investigated in future experiments.

The failure to obtain enhanced expression of endogenous GS after transfection of the β-catenin mutant S45F which is in sharp contrast to the response of the GS reporter construct may highlight another important aspect of GS expression in normal and neoplastic liver tissue. Previous work from our laboratory [19] has emphasized that expression of this enzyme is mainly controlled by mechanisms that silence expression in GS negative hepatocytes. Two possibilities which might work independently or in coordination need to be discussed. Firstly, as already noted above the factor LEF-1/TCF upon which β-catenin exhibits its influence on gene transcription is an architectural factor that in the absence of β-catenin represses the transcription of target genes [10]. Thus, the nuclear presence of β-catenin may remove LEF-1/TCF from its inhibitory position and GS can be expressed. Secondly, it was demonstrated that the negative transcription factor GSSEr-BP (glutamine synthetase silencer binding protein from rat – binding protein) that interacts with a silencer located in the first intron of the endogenous GS gene [30] is able to prevent every positive influence from the 5'-enhancer on gene expression in reporter genes ([23] and Gaunitz unpublished data). In normal hepatocytes as well as in tumour cells activation of GS (by β-catenin) is not possible as long as the silencer binding protein is present. Therefore, the interesting question remains to be resolved how this negative factor or its influence is removed or inhibited by β-catenin (Wnt-signaling).

## Methods

### Chemicals

All chemicals used were of analytical grade and if not stated otherwise were purchased from Sigma (Taufkirchen, Germany).

### Tumor samples

We analysed 52 samples of HCC from patients who had undergone operations at the Uniklinik Leipzig between 1999 and 2005. All patients provided written informed consent according to the German laws as confirmed by the local committee. After surgery the material was frozen in liquid nitrogen and kept at -80°C until use. Sections from each specimen were examined by a pathologist and classified as HCC.

### Immunohistochemical analysis

#### β-catenin detection

The surgically resected HCC specimens were fixed in 10% buffered paraformaldehyde and embedded in paraffin according to standard protocols. Analysis of β-catenin and GS expression was performed on serial sections (4 μm) from each tumor. After deparaffinization and rehydratation antigen retrieval was applied by microwaving (3 × 5 min) in citrate buffer (0.01 M sodium citrate, pH 6.0). The endogenous peroxidase was destroyed by hydrogen peroxide (3% in TBS (0.01 M Tris Base, 0.9% NaCl, pH 7.6)) and incubated for 10 min. Slides were then immersed in a solution with 0.05% avidin and 0.01% biotin (10 min) and finally incubated for 30 min in 5% goat serum (Sigma-Aldrich, Steinheim, Germany) in order to block unspecific signals. As primary antibody a β-catenin monoclonal antibody (ab610153, Transduction Laboratories, Lexington, KY) was used at a dilution of 1:90, incubated overnight at 4°C. After washing with TBS, the slides were incubated with a goat anti-mouse IgG-Fc-Biotin (abAP127B, Chemicon, USA) antibody diluted 1:1000, washed again in TBS, and incubated with POD-conjugated Extravidin (abE2886, Sigma-Aldrich, Steinheim, Germany) for 30 min. Subsequently, a POD staining with 3,3'-Diaminobenziden (DAB) (Sigma-Aldrich, Steinheim, Germany) for 7 min according to manufacturer's instructions was performed. After counterstaining with aqueous hematoxylin the dehydrated slides were covered in Roti-Histokitt (Roth, Karlsruhe, Germany).

#### Glutamine synthetase detection

Detection of glutamine synthetase was performed with the following modifications to the protocol for β-catenin: The deparaffinized and rehydrated sections were treated with 3% hydrogen peroxide and 10% goat serum. The anti GS monoclonal antibody (ab610517, Transduction Laboratories, Lexington, KY) used was diluted 1:1000 and incubated overnight at 4°C. The secondary POD-conjugated goat anti mouse IgG-FAB antibody (abA3682, Sigma-Aldrich, Steinheim, Germany) was applied at a dilution of 1:150 and incubated for 90 min. After washing the antibody reaction was visualized with DAB as substrate. A counterstaining was performed with aqueous hematoxylin before dehydration and covering using the Roti-Histokitt.

### DNA Analysis

For the isolation of DNA, 25 mg tumor tissue were used according to manufacturer's instructions (DNeasy^®^Tissue Kit, QIAGEN, Düsseldorf, Germany). The DNA sequence positions of the β-catenin and Axin1 genes were derived from NCBI reference sequences NM001904 and AF009674, respectively. To screen for mutations in the β-catenin gene (codons 5–79 of exon 3) and the Axin1 gene (codons 25–258; 378–508), genomic DNA was amplified by PCR using the following primer pair for β-catenin: 'βCAT exon 3 up' (5'-CAAT GGG TCA TAT CAC AGA TTC TT-3')/'βCAT exon 3 down' (5'-TCT CTT TTC TTC ACC ACA ACA TTT-3') and 8 pairs for the Axin1 gene: 'Axin 2/1 for' (5'-TGG TTT CAA CAG GAC AGA TTG-3') and 'Axin 2/1 rev' (5'-TCC CGG AGC AGA AAC TGT AG-3'); 'Axin 2/2 for' (5'-TAT CCA AGA GCA GGG TTT CC-3') and 'Axin 2/2 rev' (5'-GGC TTA TCC CAT CTT GGT CA-3'); 'Axin 2/3 for' (5'-GAG ACT TCG ACG GCC ACT C-3') and 'Axin 2/3 rev' (5'-TTC TCC TCG TTC GAG TCA CA-3'); 'Axin 2/4 for' (5'-GAG TCA CTG CAT TCC CTG CT-3') and 'Axin 2/4 rev' (5'-CTG GTA AAA CAT GGC AGG AT-3'); 'Axin 2/5 for' (5'-CTG GCG AGA GCC ATC TAC C-3') and 'Axin 2/5 rev' (5'-TAC AGA CTT TGG GGC TCT CC-3'); 'Axin 4 for' (5'-GGG GCC AGG AGC TCT ATT-3') and 'Axin 4 rev' (5'-TAG AGG TAA GCC TGG CTC GT-3'); 'Axin 5 for' (5'-CGC TCT TTC CCT TCA CAA AG-3') and 'Axin 5rev' (5'-CCC CAT GAA GAA CAT CAG GA-3'); 'Axin 6/1 for' (5'-TGC TTT TTG TTT TCC CCA AG-3') and 'Axin 6/1 rev' (5'-CAG GAT GCT CTC AGG GTT CT-3').

PCR was performed according to manufacturer's suggestions (Taq PCR Core Kit, Qiagen) using 100 ng of genomic DNA and 15 pmol of each primer. Cycle conditions were: 94°C for 30 s for denaturation and 55°C for 40 s for hybridization. Elongation was done at 72°C for 45 s. The enzyme was initially activated by denaturation at 95°C for 3 min and final extension was performed at 72°C for 10 min. The PCR products were checked on standard agarose gels and purified before they were sequenced.

Cycle sequencing was performed at the IZKF Core Unit Leipzig using the ABI PRISM Dye Terminator Cycle Sequencing Ready Reaction Kit (Applied Biosystems). Sequencing products were applied to an ABI prism 3100 Genetic Analyser.

### Expression plasmids for mutant forms of β-Catenin

The plasmid 'pCS2', containing the human β-catenin sequence with alanine substitutions at S33, S37, T41 and S45, was kindly provided from Dr. Kemler (Freiburg) and the plasmid designated 'pBatem' containing an N-terminal truncated version of β-catenin missing all phosphorylation sites was kindly received from Dr. Hülsken (Berlin). The plasmid 'βCATa' that was used in some experiments as control was originally constructed as a vector for the production of an anti-sense RNA for β-Catenin by introducing the β-Catenin coding region in an inverse orientation. However, this plasmid was shown to be unable to prevent Wnt-signaling by knocking-out endogeneous β-Catenin mRNA and we therefore used it for normalisation in cotransfection experiments since it has almost the same size as the expression plasmid used in these experiments.

Expression plasmids with point mutations were constructed by the introduction of these mutations by site-directed mutagenesis (Gene TailorTM Mutagenesis System, Invitrogen, Karlsruhe, Germany) according to the instructions of the manufacturer. The plasmid 'βCAT_TOTsense', containing the complete coding region for β-catenin under the control of a CMV-promoter was used as a template. The following primer pairs were used to generate the mutant β-catenin plasmids: 'H36P for' (5'-CTT ACC TGG ACT CTG GAA TCC CTT CTG GTG CCA-3') and 'H36P rev' (5'-GAT TCC AGA GTC CAG GTA AGA CTG TTG CTG-3'); 'P44A for' (5'-TCT GGT GGC ACC ACC ACA GCT GCT TCC CTG AGT-3') and 'P44A rev' (5'-ACC TTA GGT AAG ACC ACG GTG GTG GTG TCG-3'); 'S45F for' (5'-GTG CCA CCA CCA CAG CTC CTT TCC TGA GTG GCA-3') and 'S45F rev' (5'-AGG AGC TGT GGT GGT GGC ACC AGA ATG GAT-3'); 'S45P for' (5'-GTG CCA CCA CCA CAG CTC CTC CCC TGA GTG GCA-3') and 'S45P rev' (5'-AGG AGC TGT GGT GGT GGC ACC AGA ATG GAT-3').

The mutant β-catenin constructs were verified by restriction enzyme analysis and sequencing using the primer 'Mut_Control 1' (5'-TGC CCT CAT CTA GCG TCT CA-3').

### Cell lines

Cells from the line HuH7 received a DMEM medium (containing 1 g/l glucose (PAA, Cölbe, Germany)) supplemented with 10% fetal calf serum (PAA, Cölbe, Germany), 2 mmol/L glutamine and antibiotics. The cells were cultured at 37°C and 90% humidity, in an atmosphere containing 5% CO_2_. One day after seeding, the medium was removed and fresh medium was added.

### Reporter Genes

The reporter gene with the GS-5'region located between a *Hin*dIII site and an *Eco*RV site (HIII/EVpT81) controlling a reduced *herpes simplex *thymidine kinase promoter has already been described [20]. This sequence emcompasses the 5'-GS sequence from position -2520 to -2146 upstream from the transcriptional start point. The TCF reporter gene kit containing the plasmids TOP-flash and FOP-flash were purchased from Upstate Biotechnology (Lake Placid, NY).

### Transfection Experiments

For transfection HuH7 cells were cultivated at 0.25 × 10^6 ^cells per well in 6-well plates in 1.5 ml medium. Twenty four hours after the start of the culture, transfection was performed using the cationic lipid formulation Unifectin-56 (Unifect Group, Moscow, Russia) according to the manufacturer's instruction. Cells in 6-well plates were transfected with 0.5 μg DNA per well. Forty eight hours after transfection cells from 6-well plates were washed twice with Hank's solution (137 mmol/L NaCl, 5.4 mmol/L KCl, 0.4 mmol/L MgSO_4 _× 7H_2_O, 0.5 mmol/L MgCl_2 _× 6H_2_O, 0.35 mmol/L Na_2_HPO_4 _× 2H_2_O, 0.44 mmol/L KH_2_PO_4_, 2 mmol/L HEPES, pH 7.4), lysed in 100 μl of lysis buffer (77 mmol/L K_2_HPO_4_, 23 mmol/L KH_2_PO_4_, 0,2% Triton X-100, 1 mmol/L dithiothreitol, ph 7.8) and collected with a rubber policeman. Luciferase measurement was performed as described [31] using a Multilabel-Reader (Mithras LB 940, Berthold Technologies, Bad Wildbad, Germany).

## Abbreviations

GS: glutamine synthetase, HCC: hepatocellular carcinoma, TBS: Tris buffered saline, POD: peroxidase, LEF-1/TCF: lymphoid enhancer factor-1/T-cell factor, Wnt: wingless-type MMTV integration site family member, GSSEr-BP: glutamine synthetase silencer element from the rat-binding protein.

## Competing interests

The author(s) declare that they have no competing interests.
